# Molecular phylogeny of the genus *Dicronocephalus* (Coleoptera, Scarabaeidae, Cetoniinae) based on mtCOI and 16S rRNA genes

**DOI:** 10.3897/zookeys.501.8658

**Published:** 2015-04-30

**Authors:** Ga-Eun Lee, Taeman Han, Jongchel Jeong, Seong-Hyun Kim, In Gyun Park, Haechul Park

**Affiliations:** 1 Applied Entomology Division, Department of Agricultural Biology, National Academy of Agricultural Science, Jeonju 565-851, Korea; 2 Seodaemun Museum of Natural History, 25 Bangmulgwan-gil, San 5-58, Seodaemun-gu, Seoul, 120-708, Korea

**Keywords:** *Dicronocephalus*, phylogenetic relationships, *Dicronocephalus adamsi*, taxonomy, Scarabaeidae, new synonymy, Korea

## Abstract

The seven species belonging to the genus *Dicronocephalus* are a very interesting group with a unique appearance and distinct sexual dimorphism. Only one species among them, *Dicronocephalus adamsi*, has been known in the Korean fauna. This species is recognized as having a wide distribution from Tibet to Korean Peninsula and is currently represented by two subspecies that have separated geographical ranges. The phylogenetic relationships of *Dicronocephalus adamsi* were still unclear. The phylogeny of *Dicronocephalus* is reconstructed with a phylogenetic study of five species including four subspecies based on a molecular approach using mitochondrial COI and 16S rRNA genes. Our results are compared with the results obtained by previous authors based on morphological characters. They show that the tested taxa are divided into two major clades. Clade A consists of two species (*Dicronocephalus adamsi* + *Dicranocephalus yui*) and Clade B includes the others (*Dicronocephalus dabryi* + *Dicranocephalus uenoi* + *Dicranocephalus wallichii*). This result generally supports Kurosawa’s proposal except that *Dicronocephalus dabryi* and *Dicranocephalus uenoi* are newly recognized as members of a monophyletic group. We propose that *Dicronocephalus adamsi drumonti* is a junior subjective synonym of *Dicronocephalus adamsi adamsi*. These results show that three members of the *Dicranocephalus wallichii* group should be treated as species rather than subspecies. However, further research including analyses of different genetic markers is needed to reconfirm our results.

## Introduction

Genus *Dicronocephalus* Hope, 1831 is a group of medium- to large-sized beetles with a unique appearance among Cetoniinae representatives. The members of the genus show distinct sexual dimorphism such as antler-like clypeal horns and prolonged tarsomeres in males (Šípek et al. 2008). This genus is composed of seven species including nine subspecies: *Dicronocephalus adamsi adamsi* Pascoe, 1863; *Dicronocephalus adamsi drumonti* Legrand, 2005; *Dicronocephalus dabryi* (Lucas, 1872); *Dicranocephalus shimomurai* Kurosawa, 1986; *Dicranocephalus uenoi uenoi* Kurosawa, 1968; *Dicronocephalus uenoi katoi* Kurosawa, 1968; *Dicranocephalus bieti* Pouillaude, 1914; *Dicronocephalus wallichii wallichii* Hope, 1831; *Dicronocephalus wallichii bourgoini* Pouillaude, 1914; *Dicronocephalus wallichii bowringi* Pascoe, 1863; *Dicranocephalus yui yui* Kurosawa, 1968; and *Dicranocephalus yui cheni* Kurosawa, 1986 (Legrand 2005, Krajcik 2014). Geographically, the genus is widely distributed from the Himalayan foothills of Nepal to Vladivostok in Russia and to Korea, but the distribution of most species and subspecies is rather limited. In particular, *Dicranocephalus shimomurai*, *Dicranocephalus uenoi uenoi*, *Dicronocephalus uenoi katoi*, *Dicronocephalus wallichii bourgoini*, *Dicranocephalus yui yui*, and *Dicranocephalus yui cheni* are endemic to the small island of Taiwan. One species, *Dicronocephalus dabryi*, is only known in West China and Myanmar. The remaining species and subspecies are widely distributed in Asia occurring throughout the Manchuria and Indo-China (Kurosawa 1986, Šípek et al. 2008, Young 2012, Krajcik 2014).

Kurosawa (1986) proposed dividing this genus into three groups on the basis of the morphological characters: 1) the *adamsi* species-group (*Dicronocephalus adamsi*, *Dicranocephalus shimomurai*, and *Dicranocephalus yui*); 2) the *wallichii* species-group (*Dicronocephalus wallichii wallichii*, *Dicronocephalus wallichii bourgoini*, *Dicronocephalus wallichii bowringi*, and *Dicronocephalus dabryi*); and 3) the *Dicranocephalus uenoi* species-group (*Dicranocephalus uenoi*). However, he did not explain the phylogenetic relationships between these species.

Among the seven species of *Dicronocephalus*, only *Dicronocephalus adamsi* is found in the Korean fauna. This species was described from Korea, but it has been known to have a wide range across Korea, China, Tibet, and Vietnam. The range of this species is divided by a wide geographical gap between Liaoning and Shanxi provinces of China (Young 2012). Legrand (2005) divided *Dicronocephalus adamsi* into two subspecies based on this distribution pattern and morphological differences. He described populations occurring in west China as *Dicronocephalus adamsi drumonti*. This classification was accepted by Krajcik (2014), but not by Young (2012).

The subspecies of *Dicranocephalus wallichii* (*Dicronocephalus wallichii wallichii*, *Dicronocephalus wallichii bourgoini*, and *Dicronocephalus wallichii bowringi*) were originally described as valid species (Hope 1831, Pascoe 1863, Pouillaude 1914). While some authors have treated these taxa as subspecies (Paulian 1960, Mikšić 1971, 1977, Krajčík 1998, Sakai and Nagai 1998, Šípek et al. 2008, Young 2012, Krajcik 2014), some others have treated them as species (Kurosawa 1968, Devecis 2008). The controversy over whether they should be dealt with at the species or sub-species level has continued without in-depth analysis.

During a review of the genus *Dicronocephalus*, several issues were encountered, such as validation of species or subspecies rank of taxa composing *Dicronocephalus adamsi* and *Dicranocephalus wallichi* (sensu lato) and the lack of phylogenetic analysis of the genus. To resolve these questions, phylogenetic analysis was performed for the genus using *cytochrome c oxidase subunit I* (*COI*) and *16S ribosomal RNA* (*16S rRNA*) mitochondrial gene sequences as well as examination of their morphological diagnostic characters.

## Materials and methods

### Specimen sampling and examination

**Figure 1.**
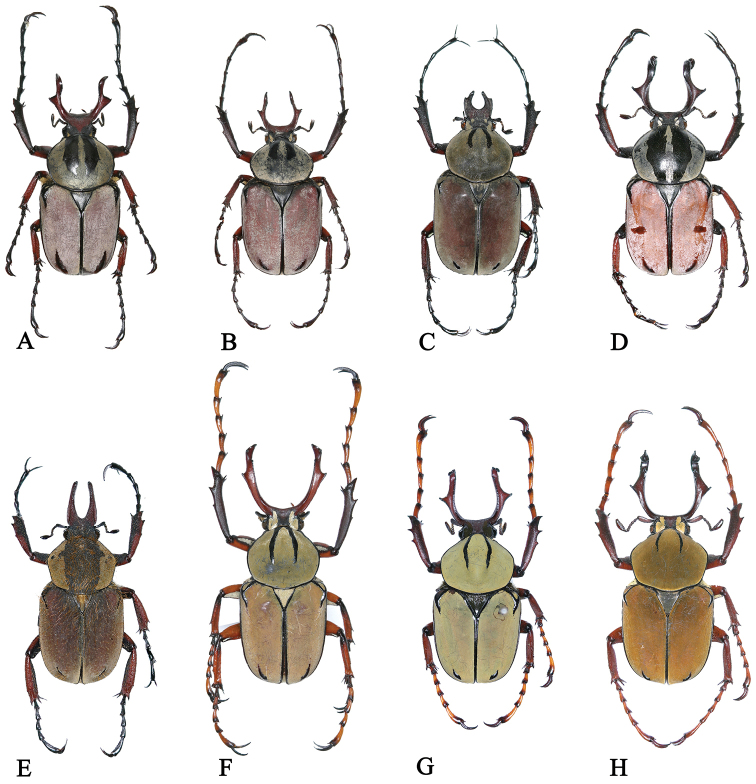
The male habitus of species and subspecies of *Dicoronocephalus*. **A** *Dicronocephalus adamsi adamsi* **B** *Dicronocephalus adamsi drumonti* **C** *Dicranocephalus yui yui* **D** *Dicronocephalus dabryi* **E** *Dicronocephalus uenoi katoi* **F** *Dicronocephalus wallichii bowringi* **G** *Dicronocephalus wallichii wallichii* **H** *Dicronocephalus wallichii bourgoini*.

Fifty specimens of *Dicronocephalus* belonging to five species and seven subspecies from four countries were obtained (Fig. 1, Table 1), but we were unable to obtain specimens of the remaining two species, *Dicranocephalus bieti* and *Dicranocephalus shimomurai*. For examining male genitalia, these were extracted from the abdomens and cleaned by heating with 10% KOH solution in a WiseTherm®HB-48P heating block at 60 °C for 1~2 hours. Male genitalia were preserved in microvials with glycerine after examination. Photographs of external morphology and genitalia were taken with a Canon EOS 10D camera and stacked with a combineZM program (Hadley 2006). Based on previous studies (Pascoe 1863, Pouillaude 1914, Kurosawa 1968, 1986, Young 2012), diagnostic characters were obtained to provide precise criteria for species identification. In this study, the most recent taxonomic scheme by Krajcik (2014) was followed, especially for subspecies treatment of *Dicranocephalus wallichii*. All examined specimens are stored in the Department of Agricultural Biology, National Academy of Agricultural Biology (NAAS), Jeonju, Korea.

**Table 1.** Collection and voucher information for specimens.

Sample no.	Species	Locality	Data collected	Sex	Voucher no.	Sequencing	
						GBAn of COI	GBAn of 16S
1	*Dicronocephalus adamsi adamsi*	Muju, JB, South Korea	6. VI. 2012	F	7258	KM390855	KM390809
2	*Dicronocephalus adamsi adamsi*	Sangdaewon-dong, Jungwon-gu, Seongnam, GG, South Korea	19. V. 2009	M	7300	KM390856	KM390810
3	*Dicronocephalus adamsi adamsi*	Sangdaewon-dong, Jungwon-gu, Seongnam, GG, South Korea	19. V. 2009	M	7301	KM390857	KM390811
4	*Dicronocephalus adamsi adamsi*	Sangdaewon-dong, Jungwon-gu, Seongnam, GG, South Korea	19. V. 2009	M	7302	KM390858	KM390812
5	*Dicronocephalus adamsi adamsi*	Sangdaewon-dong, Jungwon-gu, Seongnam, GG, South Korea	19. V. 2009	F	7303	KM390859	KM390813
6	*Dicronocephalus adamsi adamsi*	Sangdaewon-dong, Jungwon-gu, Seongnam, GG, South Korea	25. V. 2013	M	7696	KM390860	KM390814
7	*Dicronocephalus adamsi adamsi*	Sangdaewon-dong, Jungwon-gu, Seongnam, GG, South Korea	25. V. 2013	M	7697	KM390861	KM390815
8	*Dicronocephalus adamsi adamsi*	Tongrim, North Korea	VII. 1995	M	7683	KM390862	–
9	*Dicronocephalus adamsi adamsi*	North Korea	IV. 2002	M	7684	KM390863	KM390816
10	*Dicronocephalus adamsi adamsi*	Mt. Wu Long, Dandong, Liaoning, China	15. VII. 2009	M	7264	KM390864	KM390817
11	*Dicronocephalus adamsi adamsi*	Mt. Wu Long, Dandong, Liaoning, China	15. VII. 2009	M	7265	KM390865	KM390818
12	*Dicronocephalus adamsi adamsi*	Mt. Wu Long, Dandong, Liaoning, China	15. VII. 2009	M	7267	KM390866	KM390819
13	*Dicronocephalus adamsi adamsi*	Mt. Wu Long, Dandong, Liaoning, China	15. VII. 2009	M	7268	KM390867	KM390820
14	*Dicronocephalus adamsi adamsi*	Mt. Wu Long, Dandong, Liaoning, China	15. VII. 2009	M	7269	KM390868	KM390821
15	*Dicronocephalus adamsi adamsi*	Mt. Wu Long, Dandong, Liaoning, China	15. VII. 2009	M	7270	KM390869	KM390822
16	*Dicronocephalus adamsi adamsi*	Mt. Wu Long, Dandong, Liaoning, China	15. VII. 2009	M	7272	KM390870	KM390823
17	*Dicronocephalus adamsi adamsi*	Mt. Wu Long, Dandong, Liaoning, China	15. VII. 2009	M	7273	KM390871	KM390824
18	*Dicronocephalus adamsi drumonti*	Sichuan, China	VI. 2008	M	7677	KM390872	KM390825
19	*Dicronocephalus adamsi drumonti*	Sichuan, China	VI. 2008	F	7678	KM390873	KM390826
20	*Dicronocephalus adamsi drumonti*	Sichuan, China	VI. 2008	F	7679	KM390874	–
21	*Dicronocephalus adamsi drumonti*	Sichuan, China	VI. 2008	F	7680	KM390875	KM390827
22	*Dicronocephalus adamsi drumonti*	Mt. Foding, Guizhou, China	–	F	7688	KM390876	KM390828
23	*Dicronocephalus adamsi drumonti*	Tibet, China	–	M	7685	KM390877	–
24	*Dicronocephalus adamsi drumonti*	Tibet, China	–	M	7686	KM390878	KM390829
25	*Dicronocephalus adamsi drumonti*	Tibet, China	–	F	7687	KM390879	–
26	*Dicronocephalus adamsi drumonti*	Tibet, China	VIII. 2005	F	7689	KM390880	KM390830
27	*Dicronocephalus yui yui*	A- Li-Shan, Chiayi county, Taiwan	IV. 2012	F	7290	KM390881	KM390831
28	*Dicronocephalus yui yui*	A- Li-Shan, Chiayi county, Taiwan	IV. 2012	F	7291	KM390882	KM390832
29	*Dicronocephalus yui yui*	A- Li-Shan, Chiayi county, Taiwan	IV. 2012	F	7292	KM390883	KM390833
30	*Dicronocephalus dabryi*	Hanyan, Sichuan, China	16–17. VI. 2007	M	7278	KM390884	KM390834
31	*Dicronocephalus dabryi*	Hanyan, Sichuan, China	16–17. VI. 2007	M	7279	KM390885	KM390835
32	*Dicronocephalus dabryi*	H-1601m, Env. Xichang city, S. Sichuan, China	12. VI. 2009	M	7375	KM390886	KM390836
33	*Dicronocephalus dabryi*	H-1601m, Env. Xichang city, S. Sichuan, China	12. VI. 2009	F	7376	KM390887	KM390837
34	*Dicronocephalus dabryi*	China	2005	M	7690	KM390888	KM390838
35	*Dicronocephalus uenoi katoi*	Chiayi, Taiwan	VIII. 2011	M	7285	KM390889	KM390839
36	*Dicronocephalus uenoi katoi*	Chiayi, Taiwan	VIII. 2011	M	7286	KM390890	KM390840
37	*Dicronocephalus uenoi katoi*	A- Li-Shan, Chiayi county, Taiwan	IV. 2012	M	7287	KM390891	KM390841
38	*Dicronocephalus uenoi katoi*	A- Li-Shan, Chiayi county, Taiwan	IV. 2012	M	7288	KM390892	KM390842
39	*Dicronocephalus uenoi katoi*	A- Li-Shan, Chiayi county, Taiwan	IV. 2012	M	7289	KM390893	KM390843
40	*Dicronocephalus wallichii bowringi*	Mt. Lianyuan, Hunan, China	VII. 2006	M	7692	KM390894	KM390844
41	*Dicronocephalus wallichii bowringi*	Mt. Lianyuan, Hunan, China	VII. 2006	F	7693	KM390895	KM390845
42	*Dicronocephalus wallichii bowringi*	Mt. Guangwu, Sichuan, China	–	M	7694	KM390896	KM390846
43	*Dicronocephalus wallichii bowringi*	Mt. Guangwu, Sichuan, China	–	F	7695	KM390897	KM390847
44	*Dicronocephalus wallichii wallichii*	Taeng, Mae, Mai, Ching, N. Thailand	VII. 2010	M	7274	KM390898	KM390848
45	*Dicronocephalus wallichii wallichii*	Taeng, Mae, Mai, Ching, N. Thailand	IV. 2008	M	7275	KM390899	KM390849
46	*Dicronocephalus wallichii bourgoini*	Beitou, Taipei, Taiwan	V. 2008	F	7277	KM390900	KM390850
47	*Dicronocephalus wallichii bourgoini*	Beitou, Taipei, Taiwan	V. 2008	M	7280	KM390901	KM390851
48	*Dicronocephalus wallichii bourgoini*	Beitou, Taipei, Taiwan	V. 2008	M	7281	KM390902	KM390852
49	*Dicronocephalus wallichii bourgoini*	Beitou, Taipei, Taiwan	V. 2008	F	7282	198 bp	KM390853
50	*Dicronocephalus wallichii bourgoini*	Beitou, Taipei, Taiwan	V. 2008	F	7283	KM390903	KM390854
51	Protaetia brevitarsis*	Korea	–	–	–	KC775706	KC775706

* denotes outgroup taxa data extracted from GenBank. GBAn is denoted the GenBank accession number.

### DNA extraction, amplification and sequencing

Genomic DNA (gDNA) was extracted from middle legs removed from dried specimens of all species and accomplished using a QIAamp DNA Mini Kit (Qiagen, Hilden, Germany) in accordance with the manufacturer’s instructions. Polymerase Chain Reaction (PCR) was performed in order to amplify the cytochrome *c* oxidase subunit I gene (*COI*) and *16S ribosomal RNA* gene (*16S rRNA*) using Accupower PCR PreMix (Bioneer, Daejeon, Korea). The universal primer set LCO1490/HCO2198 (Folmer et al. 1994) for amplifying the DNA barcoding region (658bp) of COI sequences was not successful for all samples; this may be caused by the degraded quality of gDNA (Goldstein and Desalle 2003, Hajibabaei et al. 2006; Wandeler et al. 2007). We applied the PCR methodology for retrieving COI sequences from old specimens given in Han et al. (2014) and designed new primer pairs: LCO-Ceto232F (5’–GCHTTYCCYCGAATAAATAAYATA–3’) corresponding to HCO2198 and HCO-Ceto367R (5’–ACDGTYCADCCNGTTCCTGCNCC–3’) corresponding to LCO1490. 16S rRNA was targeted in a 600 bp region with two primers, 16SB/16SA, that successfully amplified in Lucanidae and Elateridae (Hosoya et al. 2001, Hosoya and Araya 2005, Han et al. 2009, 2010). PCR amplification conditions were as follows: for *COI*, initial denaturation at 94 °C for 5 min, then 45 cycles at 94 °C for 30 s, 46 °C for 25 s, and 72 °C for 45 s followed by a final extension at 72 °C for 3 min, and for *16S rRNA*, initial denaturation at 94 °C for 5 min, then 40 cycles at 94 °C for 1 min, 50 °C for 1 min, and 72 °C for 45 s followed by a final extension at 72 °C for 5min. The amplicons were purified using a QIA quick PCR Purification Kit (Qiagen, Hilden, Germany) after the product yield was monitored by 0.7% agarose gel electrophoresis. DNA sequencing was performed using an automated DNA sequencer (ABI 3730xl 96-capillary DNA analyzer; Applied Biosystems, Foster City, CA) with the same primers used for PCR. All sequences (excepting a 198 bp fragment of COI in no. 7282) are available from GenBank under accession numbers KM390855–KM390903 for COI and KM390809–KM390854 for 16S rRNA (Table 1).

### Phylogenetic analysis

For the phylogenetic analyses, three data sets were used, a 658 bp fragment of *COI*, 520 bp fragment of 16S rRNA sequences, and the concatenated COI and 16S rRNA sequences. The data sets were aligned using ClustalW in MEGA 5.2 (Tamura et al. 2011), and genetic distances were calculated using Kimura’s two-parameter test (Kimura 1980). The phylogenetic analyses were constructed using maximum likelihood (ML), Bayesian inference methods (BI), and maximum parsimony (MP).

ML analysis was performed with GARLI 2.0 (Zwickl 2011), and the analysis was initiated at a random start tree using GTR+I+G model parameters selected by MrModelTest (Nylander 2004), with a 10,000 generation search algorithm and 1,000 bootstrap replications. The frequencies with which to log the best score (“logevery”) and to save the best tree to file (“saveevery”) were set to 10,000 and 10,000 respectively, and the number of generations without topology improvement required for termination (“genthreshfortopoterm”) was set to 5,000. At the end of the analysis, there was no improvement in the tree topology by a log likelihood of 0.01 or better. The bootstrap values were calculated using the SumTrees program of the DendroPy package (Sukumaran and Holder 2010).

BI analysis was performed with MrBayes 3.1.2 (Ronquist and Huelsenbeck 2003). Metropolis-coupled Markov chain Monte Carlo (MCMC) analyses were run with one cold and three heated chains (temperature set to 0.2) for 5,000,000 generations and tree sampling every 100 generations. The posterior probabilities were then obtained and a majority-rule consensus tree was generated from the remaining trees after discarding the first 25% of samples.

MP analysis was performed with TNT 1.1 (Goloboff et al. 2008). The analyses, followed by tree bisection reconnection (TBR) branch swapping, used default options that performed 100 random additional sequences and saved up to ten trees per replication. To obtain the strict consensus tree, symmetric resampling (Goloboff et al. 2003) with a 33% change probability and jack-knifing with a 36% removal probability were implemented using a traditional search with 1,000 replications. Each set of results was summarized in terms of absolute frequency, and the group support values were analyzed. For bootstrap value (BP) in ML and MP, and posterior probability value (PP) in BI, supporting values of <70% as “weak”, 70–79% as “moderate”, 80–89% as “strong”, and ≥ 90% as “very strong” support were used.

## Results

### Nucleotide information for COI and 16S rRNA

The data set of *COI*, with no evidence of indel (insertion/deletion) events, had 144 (21.9%) variable sites (Vs). Of these, 140 (21.3%) were parsimoniously informative sites (PIs). The data set of *16S rRNA*, with indel events at three sites, consisted of 43 (8.3%) Vs, of which 41 (7.9%) were PIs. There was about 2.6 times more variability and the level of PIs was about 2.7 times greater in COI than in that in *16S rRNA*.

### Phylogenetic analyses of COI

**Figure 2.**
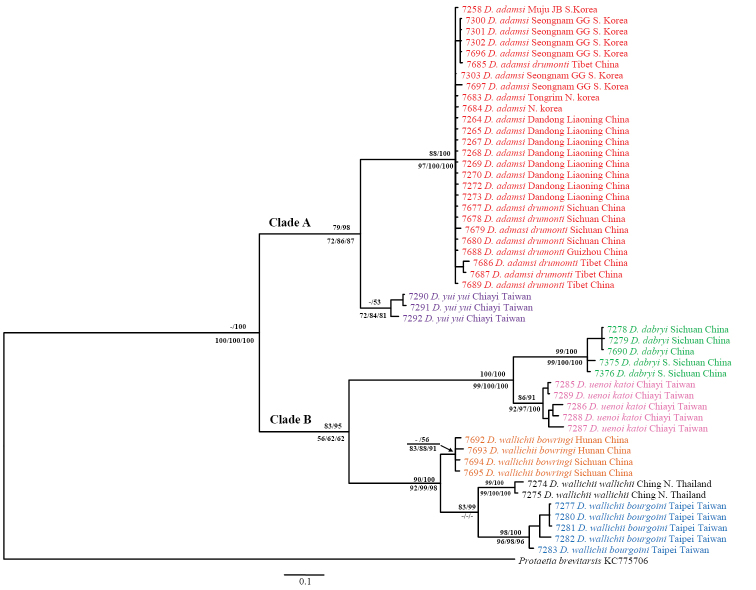
Phylogenetic relationships among *Dicronocephalus* species reconstructed with Bayesian inference using COI sequences. Numbers above branches indicate ML bootstrap values and Bayesian posterior probabilities. Numbers below branches are bootstrap, symmetric resampling, and jacknife support from parsimony searches, respectively. Scale bar represents 10% nucleotide mutation rate.

Phylogenetic inferences based on three analyses (ML, BI, and MP) reconstructed the same topologies for COI (Fig. 2; for BI, ML and MP tree data not shown, see Suppl. material 1 for sequences), and there was separation into two major clades (A and B) with very strong supporting values (100%), except for ML. Eight ingroup taxa representatives including subspecies were clearly clustered into seven monophyletic groups corresponding to nominal species; the two subspecies of *Dicronocephalus adamsi* formed one cluster. Their terminal nodes were well supported, but the values of ML and BI were very low in *Dicranocephalus yui yui* (<50% in ML and 53% in BI) and *Dicronocephalus wallichii bowringi* (<50% in ML and 56% in BI).

The intra-specific distances of COI were rather low, ranging from 0–2.3%. The inter-specific divergences were highly variable, ranging from 2.7%–16.7%. The distances between the ingroup and outgroup taxa ranged from 16.1%–20.1% (Table 2).

**Table 2.** Pairwise distance of COI within and between *Dicronocephalus* spp.

	No. of samples	Within species	Between subspecies & species						
			*Dicronocephalus adamsi adamsi* + *Dicronocephalus adamsi drumonti*	*Dicranocephalus yui yui*	*Dicronocephalus dabryi*	*Dicronocephalus uenoi katoi*	*Dicronocephalus wallichii bowringi*	*Dicronocephalus wallichii wallichii*	*Dicronocephalus wallichii bourgoini*
*Dicronocephalus adamsi adamsi* + *Dicronocephalus adamsi drumonti*	26	0.006 (0–0.017)							
*Dicranocephalus yui yui*	3	0.011 (0.002–0.017)	0.062 (0.056–0.073)						
*Dicronocephalus dabryi*	5	0.008 (0–0.015)	0.150 (0.130–0.162)	0.140 (0.130–0.149)					
*Dicronocephalus uenoi katoi*	5	0.013 (0.002–0.023)	0.150 (0.131–0.167)	0.135 (0.128–0.150)	0.069 (0.056–0.089)				
*Dicronocephalus wallichii bowringi*	4	0.006 (0.003–0.008)	0.120 (0.104–0.131)	0.117 (0.105–0.127)	0.139 (0.130–0.152)	0.117 (0.105–0.134)			
*Dicronocephalus wallichii wallichii*	2	0.006 (0.006–0.006)	0.133 (0.126–0.141)	0.123 (0.121–0.124)	0.132 (0.125–0.137)	0.135 (0.125–0.144)	0.048 (0.043–0.050)		
*Dicronocephalus wallichii bourgoini*	5	0.003 (0–0.006)	0.123 (0.109–0.134)	0.122 (0.120–0.124)	0.146 (0.131–0.163)	0.128 (0.104–0.147)	0.060 (0.048–0.081)	0.047 (0.027–0.057)	
Protaetia brevitarsis*	1	–	0.175 (0.168–0.179)	0.168 (0.164–0.170)	0.196 (0.192–0.201)	0.191 (0.188–0.196)	0.179 (0.166–0.188)	0.198 (0.197–0.199)	0.176 (0.161–0.189)

Numbers are indicated as mean (minimum-maximum) of the pairwise distance.

* denotes outgroup taxon

Clade A is composed of *Dicronocephalus adamsi adamsi*, *Dicronocephalus adamsi drumonti*, and *Dicranocephalus yui yui* with strong bootstrap support (>72%). The two subspecies of *Dicronocephalus adamsi* did not separate into two distinct subgroups. The genetic divergences between the two subspecies were relatively low (0–1.7%); moreover, *Dicronocephalus adamsi drumonti* shared haplotypes with *Dicronocephalus adamsi adamsi* from Korea and China. *Dicranocephalus yui yui* was sister to *Dicronocephalus adamsi* with distinct inter-specific divergences (5.6%–7.3%).

Clade B is composed of *Dicronocephalus dabryi*, *Dicronocephalus uenoi katoi*, and three subspecies of *Dicranocephalus wallichii* with strong bootstrap supports by ML and BI, but relatively low support (56%–62%) by MP. Among the members of Clade B, *Dicronocephalus dabryi* and *Dicronocephalus uenoi katoi* formed a monophyletic group with very strong supporting values in all analyses and with distinct inter-specific divergences (5.6%–8.9%). The intra-specific divergences of these two species (0–1.5% in *Dicronocephalus dabryi*, 0.2%–2.3% in *Dicronocephalus uenoi katoi*) were explicitly lower than their inter-specific values. The three subspecies of *Dicranocephalus wallichii* were clustered as a monophyletic group and clearly subdivided. *Dicronocephalus wallichii bowringi* diverged early from an ancestor, and then *Dicronocephalus wallichii wallichii* and *Dicronocephalus wallichii bourgoini* underwent subsequent separation with strong bootstrap supports by ML (83%) and BI (99%); however, despite low divergences within each subspecies ranging from 0.3%–0.8%, the genetic divergences between these subspecies were unexpectedly variable ranging from 2.7%–8.1%. Genetic divergences were larger between *Dicronocephalus wallichii bowringi* and both *Dicronocephalus wallichii wallichii* (4.3%–5.0%) and *Dicronocephalus wallichii bourgoini* (4.8%–8.1%), than those between *Dicronocephalus wallichii wallichii* and *Dicronocephalus wallichii bourgoini* (2.7%–5.7%).

### Phylogenetic analyses of 16S rRNA

**Figure 3.**
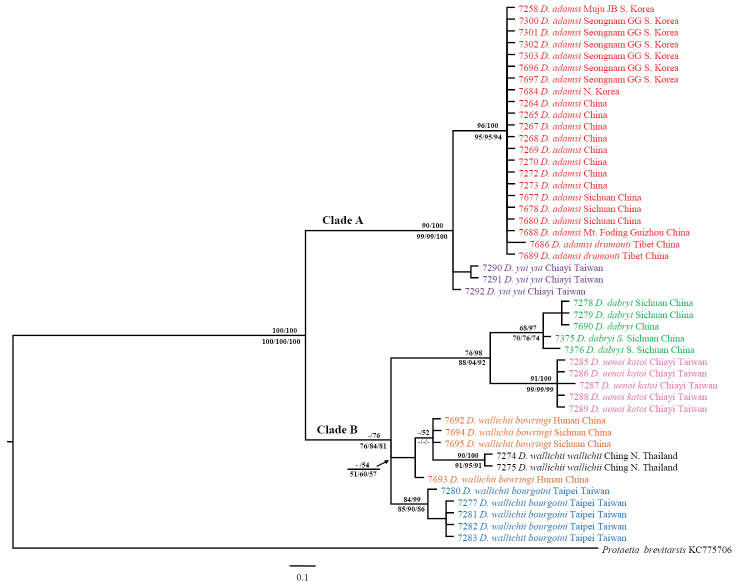
Phylogenetic relationships among *Dicronocephalus* species reconstructed with Bayesian inference using 16S rRNA sequences. Numbers above branches indicate ML bootstrap values and Bayesian posterior probabilities. Numbers below branches are bootstrap, symmetric resampling, and jacknife support from parsimony searches, respectively. Scale bar represents 10% nucleotide mutation rate.

ML, BI, and MP analyses of 16S rRNA resulted in considerably similar topologies to those of COI (Fig. 3 for BI, ML and MP tree data now shown, see Suppl. material 2 for sequences), but a polytomy was found in *Dicranocephalus yui yui* and paraphyly in *Dicronocephalus wallichii bowringi* with respect to *Dicronocephalus wallichii wallichii*.

The intra-specific pairwise distances of *16S rRNA* were relatively low, ranging from 0–0.4%. The inter-specific divergences ranged from 0.8%–6.3%. The distances between the ingroup and outgroup taxa ranged from 9.7%–11.8% (Table 3). The lowest inter-specific divergence range (0.8%–1.2%) was revealed between *Dicronocephalus adamsi* and *Dicranocephalus yui yui*, and this is rather similar to the divergence ranges of the *Dicranocephalus wallichii* subspecies (0.8%–1.6%).

**Table 3.** Pairwise distance of 16S ribosomal RNA within and between *Dicronocephalus* spp.

	No. of samples	Within species	Between subspecies & species						
			*Dicronocephalus adamsi adamsi* + *Dicronocephalus adamsi drumonti*	*Dicranocephalus yui yui*	*Dicronocephalus dabryi*	*Dicronocephalus uenoi katoi*	*Dicronocephalus wallichii bowringi*	*Dicronocephalus wallichii wallichii*	*Dicronocephalus wallichii bourgoini*
*Dicronocephalus adamsi adamsi* + *Dicronocephalus adamsi drumonti*	22	0.000 (0.000–0.002)							
*Dicranocephalus yui yui*	3	0.001 (0.000–0.002)	0.009 (0.008–0.012)						
*Dicronocephalus dabryi*	5	0.002 (0.000–0.004)	0.057 (0.054–0.060)	0.050 (0.046–0.052)					
*Dicronocephalus uenoi katoi*	5	0.001 (0.000–0.002)	0.059 (0.058–0.063)	0.052 (0.050–0.054)	0.020 (0.018–0.022)				
*Dicronocephalus wallichii bowringi*	4	0.001 (0.000–0.003)	0.046 (0.042–0.055)	0.039 (0.034–0.049)	0.035 (0.028–0.047)	0.036 (0.032–0.047)			
*Dicronocephalus wallichii wallichii*	2	0.000 (0.000–0.000)	0.050 (0.050–0.050)	0.043 (0.042–0.044)	0.030 (0.030–0.032)	0.034 (0.034–0.036)	0.009 (0.008–0.011)		
*Dicronocephalus wallichii bourgoini*	5	0.001 (0.000–0.002)	0.048 (0.048–0.048)	0.041 (0.040–0.042)	0.032 (0.028–0.034)	0.034 (0.032–0.036)	0.012 (0.008–0.016)	0.015 (0.014–0.016)	
Protaetia brevitarsis*	1	–	0.104 (0.104–0.106)	0.102 (0.101–0.104)	0.103 (0.101–0.104)	0.104 (0.104–0.104)	0.103 (0.097–0.118)	0.099 (0.099–0.099)	0.101 (0.099–0.102)

Numbers are indicated as mean (minimum-maximum) of the pairwise distance.

* denotes outgroup taxon

*Dicronocephalus adamsi* was clustered as a sister to *Dicranocephalus yui yui* in Clade A with strong bootstrap support (>90%), while the remaining taxa were clustered into Clade B with relatively low supporting values (>76%) in BI and MP. The monophyly of *Dicronocephalus adamsi*, *Dicronocephalus uenoi katoi*, *Dicronocephalus wallichii wallichii*, and *Dicronocephalus wallichii bourgoini* was well supported by bootstrap analyses (>84%). In contrast, in all analyses a polytomy was found in *Dicranocephalus yui yui* and ML and BI showed paraphyly of *Dicronocephalus wallichii bowringi*. We showed that these phenomena were caused by few parsimony-informative nucleotide variations in conserved regions. A comparison of each of those sequences, showed that *Dicronocephalus yui yui* has different substitutions at 326 nucleotide position. Two samples (7290 and 7291) have “C”, while one sample (7292) has “T”. On the other hand, *Dicronocephalus wallichii bowringi* has a substitution occurred in 196 nucleotide position. The 7693 sample has “G”, while the other samples (7692, 7694, and 7695) and two samples (7274 and 7275) of *Dicranocephalus wallichii* have “A” at this site (Suppl. material 2).

### Phylogenetic analyses of COI and 16S rRNA

**Figure 4.**
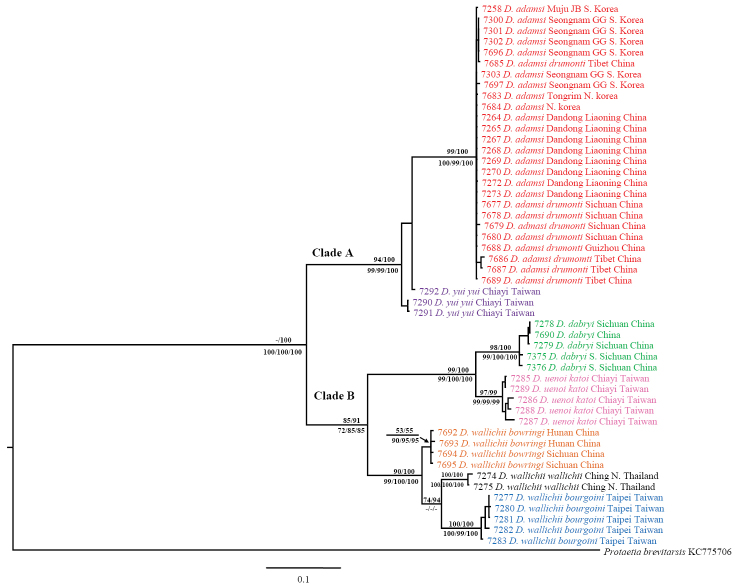
Phylogenetic relationships among *Dicronocephalus* species reconstructed with Bayesian inference using COI and 16S rRNA sequences. Numbers above branches indicate ML bootstrap values and Bayesian posterior probabilities. Numbers below branches are bootstrap, symmetric resampling, and jacknife support from parsimony searches, respectively. Scale bar represents 10% nucleotide mutation rate.

In the combined data set of COI and *16S rRNA*, phylogenetic reconstructions produced topologies congruent with the COI analyses. The nodal supporting values were improved compared with the analyses based on each gene (Fig. 4, see Suppl. material 3 for sequences). Monophyly of the seven taxa including subspecies was strongly supported by bootstrap values >90%, except for low support of 53% and 55% in ML and BI, respectively, for the terminal node of *Dicronocephalus wallichii bowringi*. *Dicronocephalus wallichii wallichii* was grouped as a sister to *Dicronocephalus wallichii bourgoini* based on the results of the COI analyses with a high value in BI (94%) and moderate value in ML (74%), but not in MP (Fig. 4).

### Re-examination of morphological diagnostic characters

**Figure 5.**
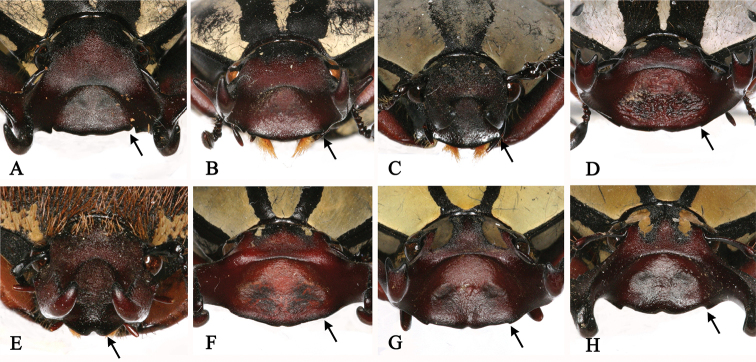
Anterior edge of clypeus of *Dicronocephalus*. **A** *Dicronocephalus adamsi adamsi* **B** *Dicronocephalus adamsi drumonti* **C** *Dicranocephalus yui yui* **D** *Dicronocephalus dabryi* **E** *Dicronocephalus uenoi katoi* **F** *Dicronocephalus wallichii bowringi* **G** *Dicronocephalus wallichii wallichii* **H** *Dicronocephalus wallichii bourgoini*.

**Figure 6.**
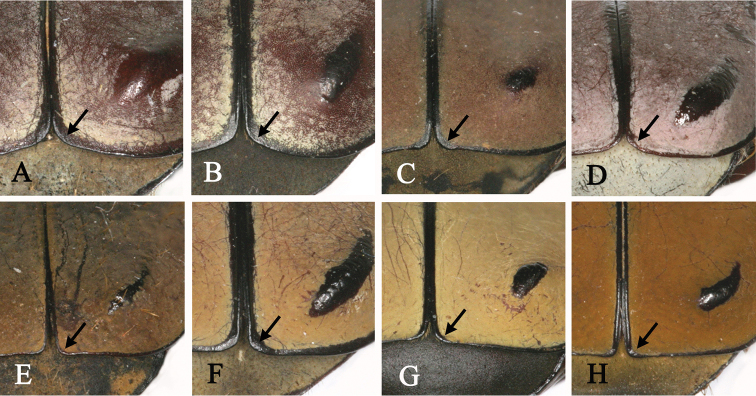
Apicosutural angle of *Dicronocephalus*. **A** *Dicronocephalus adamsi adamsi* **B** *Dicronocephalus adamsi drumonti* **C** *Dicranocephalus yui yui* **D** *Dicronocephalus dabryi* **E** *Dicronocephalus uenoi katoi* **F** *Dicronocephalus wallichii bowringi* **G** *Dicronocephalus wallichii wallichii* **H** *Dicronocephalus wallichii bourgoini*.

**Figure 7.**
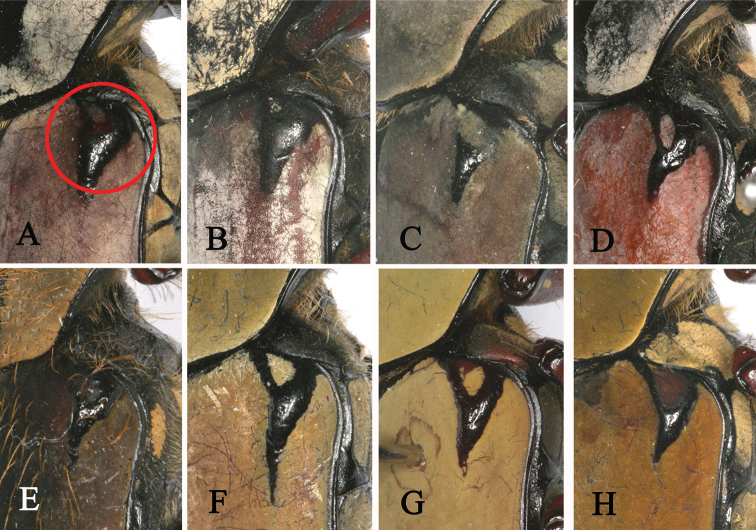
Umbone (in the circle) of shoulder of *Dicronocephalus*. **A** *Dicronocephalus adamsi adamsi* **B** *Dicronocephalus adamsi drumonti* **C** *Dicranocephalus yui yui* **D** *Dicronocephalus dabryi* **E** *Dicronocephalus uenoi katoi* **F** *Dicronocephalus wallichii bowringi* **G** *Dicronocephalus wallichii wallichii* **H** *Dicronocephalus wallichii bourgoini*.

The 19 diagnostic characters used to classify species or subspecies were re-examined in order to determine whether they are suitable for identification (Table 4). Of these characters, mentioned in previous studies, 13 are clearly suitable for species or subspecies identification; however, we recognized six characters that are ambiguous and not applicable (Table 5). For example, Pouillaude (1914) mentioned three diagnostic characters as follows: 1) *Dicronocephalus dabryi* has a different color of the pronotum and the elytra compared with *Dicranocephalus wallichii* subspecies (Fig. 1); 2) *Dicronocephalus wallichii wallichii* can be separated from the others (*Dicronocephalus adamsi*, *Dicronocephalus wallichii bowringi*, *Dicronocephalus wallichii bourgoini*, *Dicronocephalus dabryi*, and *Dicranocephalus beiti*) by having no angular projection at the base of the anterior edge of the clypeus (Fig. 5); and 3) *Dicronocephalus wallichii bourgoini* can be distinguished from the others by the projected apicosutural angle of the elytra (Fig. 6). However, none of these characters has proven to be suitable for species identification. We observed that the color of the pronotum and the elytra of *Dicronocephalus dabryi* was the same with grayish powder in freshly collected specimens, but it has faded gradually in old specimens (Fig. 1D). Also the anterior edge of the clypeus of *Dicronocephalus wallichii wallichii* (Fig. 5G) was sinuate in the middle, similar to that of *Dicronocephalus wallichii bourgoini* (Fig. 5H), and did not match the description by Pouillaude. We therefore consider that these characters might have been mistakenly described and illustrated by Pouillaude (1914). In addition, the projection of the apicosutural angle of the elytra of *Dicronocephalus wallichii bourgoini* was not distinct and could not separate this taxon from the other species and subspecies (Fig. 6H). We consider that using another character such as “the posterior margin of the elytra is round or truncated” may more diagnostic than the former character as shown in Fig. 6. Pascoe (1863) used the triangular umbone on the shoulder of the elytra (Fig. 7) to distinguish *Dicronocephalus adamsi adamsi* from *Dicronocephalus wallichii bowringi*. But, we consider that the presence of a triangular umbone is as an unsuitable character. We found this state also in some specimens of *Dicronocephalus adamsi*, although the size of the triangular umbone was small and variable in each specimen. Kurosawa (1986) used the widest portion of the pronotum as a distinguishing character state, but this was variable in all specimens of *Dicronocephalus wallichii bourgoini* and not distinct enough to be used in species and subspecies identification.

**Table 4.** Diagnostic characters of ***Dicronocephalus***.

Character		states	Reference
Body	1. Color in male (Fig. 1)	0) grayish brown	Kurosawa (1968)
		1) dark brown	
		2) yellowish brown	
		3) dark yellowish brown	
		4) green-yellowish brown with pale purple on elytra	
	2. Color in female	0) dark blackish body without marking	Kurosawa (1986)
		1) not dark blackish body	
	3. Pronotal and elytral colors (Fig. 1)	0) pronotum and elytra different	Pouillaude (1914)
		1) pronotum and elytra similar	
	4. Dorsal surface	0) pilose with brownish semirecumbent hairs	Pouillaude (1914) Kurosawa (1968)
		1) almost hairless	
		2) sparsely pilose with hair	
Head	5. Development of antlers	0) a pair of antlers in male very short, undeveloped, approximate to each other anteriorly	Kurosawa (1968)
		1) antlers in male long and well developed, curving upwards apically and broadly separated from each other	
	6. Inferior dentation of antlers	0) clearly projected upward	Kurosawa (1968)
		1) weakly prominent	
		2) absent	
	7. Shape of anterior edge of clypeus (Fig. 5)	0) simple without angular projection	Pouillaude (1914)
		1) with an angular projection	
	8. Circular indentation of clypeus	0) with a strong or weak circular indentation on the edge	Pouillaude (1914)
		1) without circular indentation on the edge	
Pronotum	9. Pronotal bands	0) reaching posterior border	Pouillaude (1914) Young (2012)
		1) not reaching posterior border	
	10. Central carinae	0) carinae defined	Pascoe (1866)
		1) carinae nearly indistinct	
	11. Extending of carinae	0) extending beyond the middle	Kurosawa (1968)
		1) never extending beyond the middle	
		2) no carina	
	12. The widest portion	0) widest near the middle	Kurosawa (1968)
		1) widest in front of the middle	
Elytra	13. Surface	0) with two black dots	Young (2012)
		1) without black dot	
	14. Shoulder (Fig. 6)	0) with triangular umbone	Pascoe (1866)
		1) without triangular umbone	
	15. Apicosutural angle (Fig. 7)	0) rounded	Pouillaude (1914)
		1) projected	
Metasternum	16. Metasternal process	0) obtuse, rather rounded	Kurosawa (1968) Young (2012)
		1) rectangular or acute, moderately produced	
		2) triangularly and sharply produced	
Abdomen	17. Abdominal sternites in male	0) covered with yellowish grey powder	Pouillaude (1914)
		1) normal, not covered with yellowish grey powder	
Legs	18. Color of tarsi	0) clear reddish brown (=testaceous)	Pascoe (1866) Pouillaude (1914) Young (2012)
		1) black or very dark brown	
	19. Length of tarsi	0) anterior tarsi of the male about as long as posterior ones	Kurosawa (1968)
		1) anterior tarsi distinctly longer than the others	

**Table 5.** Data matrix for *Dicronochephalus* species in this study.

	1	2	3	4	5	6	7	8	9	10	11	12	13	14	15	16	17	18	19
*Dicronocephalus adamsi adamsi*	**0**	0	**1**	**1**	**1**	?	1	0	1	1	**2**	**0**	**1**	1 (rarely 0)	0	**1**	0	1	**1**
*Dicronocephalus adamsi drumonti*	**0**	**0**	**1**	**1**	**1**	?	**0**	**0**	**1**	**1**	**2**	**0**	**1**	**1 (rarely 0)**	**0**	**1**	**0**	**1**	**1**
*Dicranocephalus yui yui*	**1**	0	**1**	1	0	**2**	**0**	**0**	**1**	**1**	**1**	0 (or 1)	**1**	**1 (rerely 0)**	?	1	**0**	**1**	1
*Dicronocephalus dabryi*	**0**	1	0 (or 1)	1	**1**	?	1	1	0	**1**	**2**	**1**	0	**1 (rarely 0)**	0	0	1	1	**1**
*Dicronocephalus uenoi katoi*	1	**1**	**1**	0	0	**2**	**1**	**0**	**0**	**1**	**2**	**1**	**1**	**1**	?	0	**1**	**1**	0
*Dicronocephalus wallichii bowringi*	**3**	1	1	**1**	**1**	0 (or 1)	0 (or 1)	1	1	0	0	**0**	**1**	0	0	**1**	1	0	**1**
*Dicronocephalus wallichii wallichii*	**2**	1	1	**1**	**1**	**0**	0 (or 1)	1	1	0	0	**0**	**1**	**0**	0	2	1	0	**1**
*Dicronocephalus wallichii bourgoini*	4	1	1	1	1	1	1	1	1	**0**	1	1 (rarely 0)	**1**	**0**	1	1	1	0	1
Results of examination	C	C	U	C	C	U	U	C	C	C	C	U	C	U	U	C	C	C	C

Boldic numbers indicate additionally examined diagnostic characters at each species in this study. Parentheses denote the characteristic represeted by our examination. Question marks indicate the ambiguous character state to be difficult determination in our examination. ‘C’ is clear and ‘U’ is unclear characters resulted in this study.

**Figure 8.**
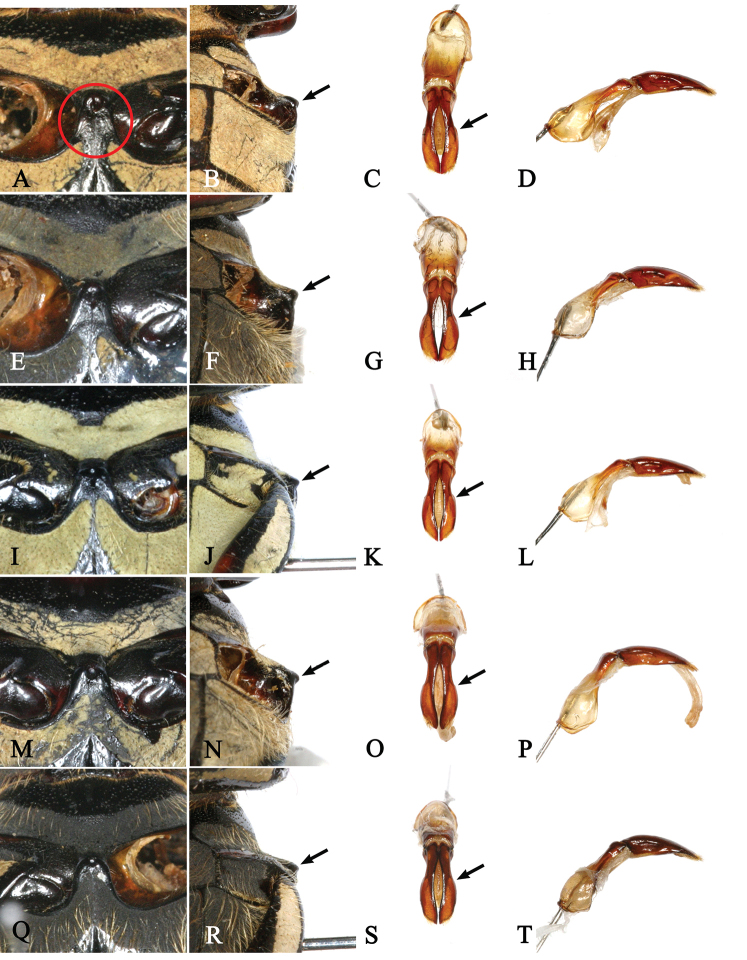
Metasternal process (in the circle) and aedeagi of *Dicronocephalus adamsi drumonti* and *Dicronocephalus adamsi adamsi*. **A, B, C, D** *Dicronocephalus adamsi drumonti* (Tibet) **E, F, G, H** *Dicronocephalus adamsi drumonti* (Sichuan) **I, J, K, L** *Dicronocephalus adamsi adamsi* (South Korea) **M, N, O, P** *Dicronocephalus adamsi adamsi* (North Korea) **Q, R, S, T** *Dicronocephalus adamsi adamsi* (Dandong, China).

Legrand (2005) used six diagnostic characters to distinguish between the two subspecies, *Dicronocephalus adamsi adamsi* and *Dicronocephalus adamsi drumonti*. Among them, we found four characters, namely body size, general body shape, longitudinal bands on the pronotum, and the shape of the triangular umbone of the elytra, to be ambiguous. He also illustrated the metasternal process and the parameres and explained in the key to subspecies that the ridge of the metasternal process does not reach the plate, and the process is weakly raised and more rounded anteriorly in *Dicronocephalus adamsi drumonti*. Also, the parameres of *Dicronocephalus adamsi drumonti* are shorter and with more acute lateral angles than of *Dicronocephalus adamsi adamsi*. However, we found that these characters were variable in the specimens from the two geographically isolated populations (Fig. 8). For example, the shape of the lateral angles of the parameres of Tibetan *Dicronocephalus adamsi drumonti* (Fig. 8C, D) is similar to that of a *Dicronocephalus adamsi adamsi* from South Korea (Fig. 8K, L), and another specimen of *Dicronocephalus adamsi drumonti* from Sichuan, China (Fig. 8G, H) resembles a *Dicronocephalus adamsi adamsi* from Dandong, China (Fig. 8S, T). We did not find any significant diagnostic characters to separate the two subspecies and therefore the new synonymy is here proposed (*Dicronocephalus adamsi drumonti* Legrand, 2005 = *Dicronocephalus adamsi adamsi* Pascoe, 1863, syn. nov).

## Discussion

From the results inferred from ML, BI, and MP methods using COI and 16S rRNA genes, the genus *Dicronocephalus* includes two major lineages, one with *Dicronocephalus adamsi* and *Dicranocephalus yui yui* and another with *Dicronocephalus dabryi*, *Dicronocephalus uenoi katoi*, *Dicronocephalus wallichii bowringi*, *Dicronocephalus wallichii wallichii*, and *Dicronocephalus wallichii bourgoini* (Figs 1–3). The specimens of eight taxa including subspecies clustered into seven groups and their monophyly was strongly supported in all analyses. However, *Dicronocephalus wallichii bowringi* was found to be paraphyletic and the monophyly of *Dicranocephalus yui yui* was not confirmed in the 16S rRNA based analyses. In the same analyses we also failed to identify the monophyly of *Dicranocephalus yui yui* (Fig. 3). Paraphyly or polytomy of the two species was the result of a few pasimony-informative nucleotide substitutions. This has a significant effect on phylogenetic reconstructions when the genetic divergences within and between species are low.

In all topologies, *Dicronocephalus adamsi* is sister to *Dicranocephalus yui yui*; the same was suggested by Kurosawa (1986). He grouped *Dicronocephalus adamsi*, *Dicranocephalus shimomurai*, and *Dicranocephalus yui* as the *adamsi* species-group and mentioned that the female dark blackish body without markings might be the main characteristic of this group. The abdomen covered with whitish powder is also a trait that is only shared by *Dicronocephalus adamsi* and *Dicranocephalus yui* among the examined species (Pouillude 1914, Kurosawa 1986).

In contrast with the molecular data of the *adamsi* species-group, our results for the other congeners do not support the view of Kurosawa (1986). *Dicronocephalus uenoi katoi* is treated as a separate group in his paper, but it appears a sister taxon of *Dicronocephalus dabryi* in our study, although the general appearance of *Dicronocephalus uenoi katoi* is rather similar to that of *Dicranocephalus yui yui*. Especially, these two species share two characters: the pronotal bands reaching the posterior border and the obtuse metasternal process. Pouillaude (1914) also noted that *Dicronocephalus dabryi* has tawny erect hair on the pronotum and elytra. We could observe that the pronotum and elytra are sparsely pilose and the hairs are much denser and longer on the ventral side compared with the other congeners. Furthermore, in the male genitalia, the parameres of the two species are similar and much shorter than those of other species. In this study, the pilose body, which is represented as a unique character of *Dicronocephalus uenoi katoi* by Kurosawa (1986), is considered as autapomorphy, which may have been rapidly acquired during allopatric speciation in Taiwan because *Dicronocephalus uenoi katoi* was isolated from a continental ancestor. This interpretation disagrees with Kurosawa’s presumption that *Dicronocephalus uenoi katoi* is the most primitive in this genus.

Regarding the status of the subspecies of *Dicronocephalus adamsi*, Legrand (2005) recognized discontinued distribution and morphological differences between two geographically separated populations; however, we consider almost all of the diagnostic characters as being unsuitable for distinguishing these two subspecies. Furthermore, the molecular data indicates that the two subspecies form a monophyletic group with low genetic divergences (0–1.7%) and individuals of the both subspecies share haplotypes. Therefore, our results provide strong evidence that *Dicronocephalus adamsi drumonti* should be synonymized with *Dicronocephalus adamsi adamsi*.

The three subspecies of *Dicranocephalus wallichii* were originally described as separate species (Hope 1831, Pascoe 1863, Pouillaude 1914). Subsequently their status was lowered to subspecific (Paulian 1960, Mikšić 1971, 1977, Krajcik 1998, Sakai and Nagai 1998, Šípek et al. 2008, Young 2012, Krajcik 2014). However, Kurosawa (1968) disagreed with Paulian (1960) as he considered that there were significant morphological differences between them such as the characteristics of the antlers, the clypeus, the marginal carinae of the pronotum, and the metasternal process. Devecis (2008) also proposed that the taxa be restored as species based on the morphological differences such as color of the dorsal setation, shape of the antlers, and length of the pronotal bands. Results of our molecular analyses showed that the three subspecies of *Dicranocephalus wallichii* form a monophyletic group with high supporting values and large genetic distances. The average pairwise distances (4.7%–6.0%) of COI between *Dicronocephalus wallichii bowringi* + *Dicronocephalus wallichii wallichii* and *Dicronocephalus wallichii bowringi* + *Dicronocephalus wallichii bourgoini*. *Dicronocephalus wallichii wallichii* + *Dicronocephalus wallichii bourgoini* were slightly lower than the average inter-specific distances of *Dicronocephalus adamsi* + *Dicranocephalus yui yui* (6.2%) and *Dicronocephalus dabryi* + *Dicronocephalus uenoi katoi* (6.9%) (Table 2). Also, in *16S* rRNA analysis, the pairwise distances between the three subspecies of *Dicranocephalus wallichii* were similar to (0.8%–1.6%) the distance between *Dicronocephalus adamsi* and *Dicranocephalus yui yui* (0.8%–1.2%) (Table 3). Our phylogenetic analyses explicitly explain their evolutionary history. *Dicronocephalus wallichii bowringi* is the most primitive among this group and *Dicronocephalus wallichii wallichii* might be separated by parapatric speciation in the continental region. Also, *Dicronocephalus wallichii bourgoini* might have undergone allopatric speciation after colonizing the volcanic island of Taiwan. Our results support specific rather than subspecific rank of the three members of *Dicranocephalus wallichii*. We revealed them as being in a monophyletic cluster (Mishler and Theriot 2000, Wiens and Penkrot 2002) with each other separated by distinct genetic gaps in the COI and *COI*+*16S* analyses, although not in the 16S rRNA analysis. Also, our study showed two distinguishable morphological characters, namely the color of the dorsal body side in males and the shape of the metasternal process (Table 5). However, this evidence is not strong enough to propose specific rank for each of them. A recent study showed that the high genetic divergence of COI alone cannot be a reason for species separation in *Cetonia aurata aurata* (Ahrens et al. 2013). There is a need for additional analyses with representative sample sizes and the use of multiple genetic loci to reconfirm our results.

## Supplementary materials

Supplementary material 1 *COI* sequences dataset of *Dicronocephalus* species in this study. Data type: (DNA sequences) Explanation note: This COI data includes 50 individual sequences of the examined *Dicronocephalus* species and subspecies in this study This dataset is made available under the Open Database License (http://opendatacommons.org/licenses/odbl/1.0/). The Open Database License (ODbL) is a license agreement intended to allow users to freely share, modify, and use this Dataset while maintaining this same freedom for others, provided that the original source and author(s) are credited.Ga-Eun Lee, Taeman Han, Jongchel Jeong, Seong-Hyun Kim, In Gyun Park, Haechul Park

Supplementary material 2 *16S rRNA* sequences data set of *Dicronocephalus* species in this study. Data type: (DNA sequences) Explanation note: This 16S rRNA data includes 46 individual sequences of the examined *Dicronocephalus* species in this study. This dataset is made available under the Open Database License (http://opendatacommons.org/licenses/odbl/1.0/). The Open Database License (ODbL) is a license agreement intended to allow users to freely share, modify, and use this Dataset while maintaining this same freedom for others, provided that the original source and author(s) are credited.Ga-Eun Lee, Taeman Han, Jongchel Jeong, Seong-Hyun Kim, In Gyun Park, Haechul Park

Supplementary material 3 The combined dataset of COI and 16S rRNA of *Dicronocephalus* species in this study. Data type: (DNA sequences) Explanation note: There is the concatenated sequences of COI and 16S rRNA genes correspondence with each sample. This dataset is made available under the Open Database License (http://opendatacommons.org/licenses/odbl/1.0/). The Open Database License (ODbL) is a license agreement intended to allow users to freely share, modify, and use this Dataset while maintaining this same freedom for others, provided that the original source and author(s) are credited.Ga-Eun Lee, Taeman Han, Jongchel Jeong, Seong-Hyun Kim, In Gyun Park, Haechul Park
